# Spring arctic oscillation as a trigger of summer drought in Siberian subarctic over the past 1494 years

**DOI:** 10.1038/s41598-021-97911-2

**Published:** 2021-09-24

**Authors:** Olga V. Churakova Sidorova, Rolf T. W. Siegwolf, Marina V. Fonti, Eugene A. Vaganov, Matthias Saurer

**Affiliations:** 1 Institute of Ecology and Geography, Siberian Federal University, Svobodniy pr. 79/4, 660041 Krasnoyarsk, Russia; 2 Swiss Federal Institute for Forest, Snow and Landscape Research WSL, Zürcherstrasse 111, 8903 Birmensdorf, Switzerland; 3 Siberian Federal University, Rectorate, Svobodniy pr 79/10, 660049 Krasnoyarsk, Russia; 4 Sukachev Institute of Forest SB RAS, Federal Research Center “Krasnoyarsk Science Center SB RAS”, Akademgorodok 50, bld. 28, 660036 Krasnoyarsk, Russia

**Keywords:** Biogeochemistry, Climate sciences, Ecology, Environmental sciences

## Abstract

Rapid changes in the hydrological and temperature regimes over the past decades at the northern latitudes enhance significantly permafrost degradation accelerating carbon release, increase the frequency of drought events and extensive wildfires. However, the mechanisms and dynamics driving drought events and their influence on Siberian forests are currently the subject of numerous research activities. Newly developed and annually resolved stable carbon and oxygen isotope chronologies of larch tree-ring cellulose (δ^13^C_cell_ and δ^18^O_cell_) for the period 516–2009 CE allowed the reconstruction of July precipitation and Arctic Oscillation (AO) in May, respectively. Unprecedented drought events occurred towards twentieth–twenty-first centuries as indicated by the July precipitation reconstruction. Positive AO phases in May were most pronounced during the second part of the first millennium, but also increased in frequency in the modern period of the twentieth–twenty-first centuries. Negative AO phases are associated with cold anomalies and show a remarkable decrease in the nineteenth century caused by a series of major volcanic eruptions. Our findings help explaining the increased frequency of Siberian forest fires over the past decades in Central Siberia consistent with a reduction of summer precipitation, triggered by a positive phase of the Arctic Oscillation in May.

## Introduction

Over the past two decades, heatwaves occur more often in the Eurasian north and particularly in the Siberian subarctic. Larch forests growing in these regions are highly sensitive to climatic changes due to severe environmental conditions^1–3^. Recent rapid changes in the hydrological and temperature regimes affect significantly permafrost degradation^4^ accelerating carbon release, increase frequency of drought events and extensive wildfires expanded over a large territory in Siberia^5–8^. However, the mechanisms leading to drought events in the permafrost region with available thawed water for Siberian forests remain unknown.

Siberian weather stations observation covers mainly the last 100 years, with the best quality data back to 1966 CE only. To obtain information about past climatic changes it is, therefore, necessary to consider indirect climatic archives like tree rings^9,10^. Tree rings have been proven to be a valuable tool for climate reconstructions from the Eurasian subarctic back in time up to several millennia^11–14^. Tree-ring width chronologies from the Siberian subarctic record mainly June-July air temperature signal^11–13^. The variation of stable carbon isotopes in tree rings can provide complementary information about changes in precipitation^15–19^, sunshine duration^20^, vapor pressure deficit^21^ and cloud cover^22^, while oxygen isotopes can record information about atmospheric circulation patterns^16,23–27^.

The Arctic Oscillation (AO) has an impact on winter temperature and precipitation patterns over Eurasia and North America^28^ and particularly in Siberia^29^. A positive AO phase (AO+) indicates higher pressure at mid-latitudes, which brings higher than average temperatures to northern Eurasia, leading to enhanced greening conditions over large regions, which is confirmed by satellite-derived vegetation indices^30^. A study by Baltzer et al.^31^ showed a significant relationship between annual burned forest areas in Central Siberia and AO, while Kim et al.^7^ showed that positive Arctic Oscillation (AO) phases lead to an increasing fire activity in Asian regions. A high-pressure of the AO circulation was related to a high-temperature anomaly in late winter and spring during the past decade^32^.

Opposite to the AO+, a negative AO brings cold weather anomaly, storms, and wetter conditions at the high-latitude regions^28^. Reconstructions of AO may, therefore, provide information on past temperature and precipitation patterns with annual and seasonal resolutions.

To apply any measure and mitigation strategies to decrease carbon release in Siberia under rapid temperature and hydrological changes, it is urgent to obtain information about fluctuation rates of climatic changes over the past. To reach this goal we built continuous annually-resolved stable carbon and oxygen isotope chronologies from larch tree rings and used multiple linear correlation analysis to test for the driving factors leading to precipitation changes in Siberian forests over the past 1494 years.

## Results

### Millennial stable carbon and oxygen isotope chronologies

**Figure 1:**
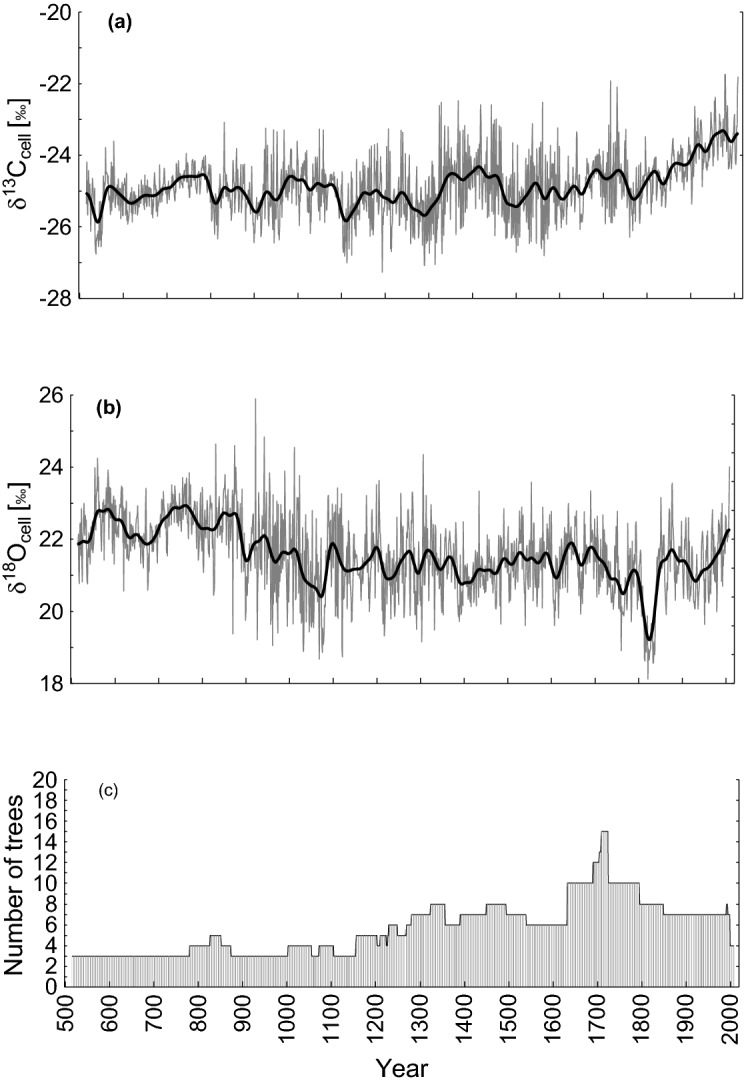
Annually resolved (grey) and smoothed by a 41-year Hamming window (bold black) stable carbon δ^13^C_cell_ (**a**) and oxygen (δ^18^O_cell_) (**b**) isotope chronologies. Number of trees (**c**) used for both δ^13^C_cell_ and δ^18^O_cell_ chronologies.

The δ^13^C and δ^18^O tree-ring cellulose (δ^13^C_cell_ and δ^18^O_cell_) chronologies based on the 42 larch trees for the period from 516 to 2009 CE were developed (Fig. 1). A clear increase in the δ^13^C_cell_ trend accelerating towards recent decades is detected (Fig. 1a), which is not the case for the δ^18^O_cell_ (Fig. 1b).

The increasing trend in δ^13^C_cell_ starts in the 1800s (Fig. 1a). The maximum values and high variability of δ^13^C_cell_ were detected mainly for the twentieth century, specifically in 1979 (− 21.7‰; + 3.9σ), 1980 (+ 3.9σ) and 2009 CE (+ 3.8σ) (Supplementary Table S1a) in relation to the mean (− 24.9‰, SD = 0.8, SE =  ± 0.02) for the whole study period from 516 to 2009 CE (Supplementary Table S1a). Low δ^13^C values were found during the twelfth-fourteenth centuries, with a minimum value in 1194 CE (− 27.3 ‰; − 3σ).

Maximum δ^18^O values were recorded during the tenth and twenty-first centuries, with most extreme values in the years of 923 CE (25.9‰; 4.2σ) and 2009 CE (24.01‰; 3.8 σ). Minimum δ^18^O values were recorded during the eleventh, twelfth and nineteenth centuries with the minimum value in 1822 CE (18.1‰; − 3.3 σ) compared to the average (21.6‰. SD = 1.04, SE ± 0.03) over the whole analyzed period (Supplementary Table S1b).

#### Stable isotopes vs. climatic parameters

**Figure 2:**
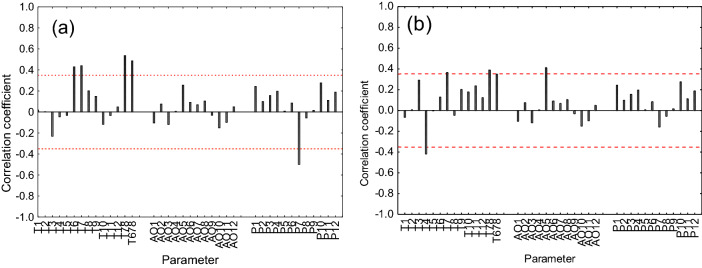
Correlation coefficients calculated between climatic parameters (temperature—T; Arctic Oscillation index—AO and precipitation—P) and the δ^13^C_cell_ (**a**) and δ^18^O _cell_ (**b**) chronologies over the common period of observations from 1969 to 2009 CE. Dashed red lines represent a level of significance at *P* < 0.05. Numbers from 1 to 12 indicate months.

Significant Pearson correlations (*P* < *0.001*) were revealed between summer air temperature [June, July, averaged July–August and June–July–August (JJA)] and δ^13^C_cell_ (Fig. 2a) as well as δ^18^O _cell_ (Fig. 2b). Positive significant correlations were found between δ^13^C_cell_ and June (r = 0.43), July (r = 0.44), averaged July–August (r = 0.54), JJA (r = 0.49) temperatures, respectively, while a negative significant correlation between δ^13^C_cell_ and July precipitation (r = − 0.50, *P* < 0.001) was revealed (Fig. 2a). Less pronounced was the summer air temperature signal in δ^18^O_cell_, expressed in marginally significant relationships between δ^18^O_cell_ and July air temperature (r = 0.36, *P* < *0.05*), July–August (r = 0.39) and JJA (r = 0.35), respectively (Fig. 2b). A negative correlation with April temperature was found with the δ^18^O_cell_. The strongest influence on δ^18^O_cell_ was AO index. The AO in May correlates significantly with δ^18^O_cell_ (r = 0.38) for the common period 1969 to 2009 CE.

#### July precipitation reconstruction

**Figure 3:**
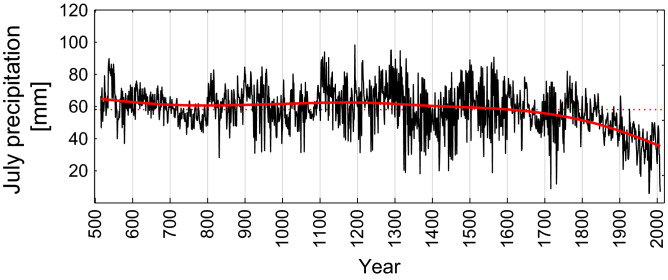
A high-frequency July precipitation reconstruction derived from the δ^13^C_cell_ (black line) and fitted distance weighted LS (bold red line). The standard deviation (SD = 13.39 mm/July, SE =  ± 0.35) analyzed for the period 516–2009 CE is represented by the dashed red line.

Based on the correlation (Fig. 2) and multiple linear regression (Eq. 2) analyses, and calibration and verification statistics (*P* < 0.0001), July precipitation reconstruction was computed for the period from 516 to 2009 CE (Fig. 3, Supplementary Fig. S2, Supplementary Table S2).

The average amount of precipitation for the period from 516 to 2009 CE is 58.15 mm (SE =  ± 0.19). A significant decrease of July precipitation from 60.4 mm for the period 516–1799 CE (r^2^ = 0.01, *P* < 0.05) to 44.3 mm for the period 1800–2009 CE (r^2^ = 0.46, *P* < 0.001) was observed (Fig. 3). Several periods characterized by low amounts of July precipitation, particularly during the sixth, ninth–tenth, thirteenth–sixteenth, eighteenth and the twentieth–twenty-first centuries, were revealed (Table 1). High amounts of July precipitation are recorded in the late 6th, 12th and the sixteenth centuries with the maximum wet extreme in 1194 CE relative to the whole study period 516–2009 CE (Table 1). A reduction in July precipitation is clearly observed since the early nineteenth century.

**Table 1:** Warm and cold extremes were reconstructed based on the δ^18^O_cell_ for Arctic Oscillation (AO) in Mai positive (AO+) and negative (AO−) phases respectively. Extremely dry and wet years of July precipitation were derived from the δ^13^C_cell_ chronology from Taimyr Peninsula over the past 1494 years. As reference major volcanic eruptions with volcanic explosivity index^33^ (VEI ≥ 4) and wildfires reported from other studies^5,31,34,35^ were considered. Extrem years (≥ − 3σ) are marked as asterick.

Arctic Oscillation (AO) index (δ^18^O_cell_)		July precipitation (δ^13^C_cell_)		Extreme climatic events	
Negative phase (AO−) Extremely cold years ≥ − 2σ	Positive phase (AO+) Extremely warm years ≥ + 2σ	Positive phase Extremely wet years ≥ + 2σ	Negative phase Extremely dry years ≥ − 2σ	Years of major volcanic eruptions, Volcanic Explosivity Index^33^ (VEI ≥ 4)	Wildfire years^5,31,34,35^
871, 927, 962, 974, 1032–1033, 1069, 1075–1079, 1121, 1122, 1304, 1761, 1814–1815, 1818–1820, 1822*–1828, 1831, 1834–1838	561, 832, 875, 923*, 943, 1012, 1307, 943*, 955, 990, 1012, 1024, 1307, 2009	537, 538, 544, 550, 1104–1106, 1108, 1113, 1118, 1124, 1194*, 1213, 1215, 1259, 1286, 1290, 1291, 1302, 1315, 1331, 1501, 1505, 1559, 1565	832, 927, 1050, 1208, 1325, 1329, 1337, 1345, 1368, 1383, 1419, 1420, 1444, 1448, 1451, 1455, 1462, 1466, 1538, 1561, 1596, 1713, 1717*, 1732*, 1733, 1910, 1917, 1923–1925, 1947–1949, 1955, 1956, 1957*,1958, 1968, 1969, 1975, 1979*, 1980*, 1981, 1984, 1985, 2000, 2001, 2006, 2007, 2008*, 2009 *	536, 541, 1257, 1812, 1815, 1822	1700–1740, 1770–1795, 1860–1895, 1923, 1953–1955, 1924, 1978, 1979, 1980, 1984, 2002, 2008, 2009

#### Reconstructed arctic oscillation (AO) index in May

**Figure 4:**
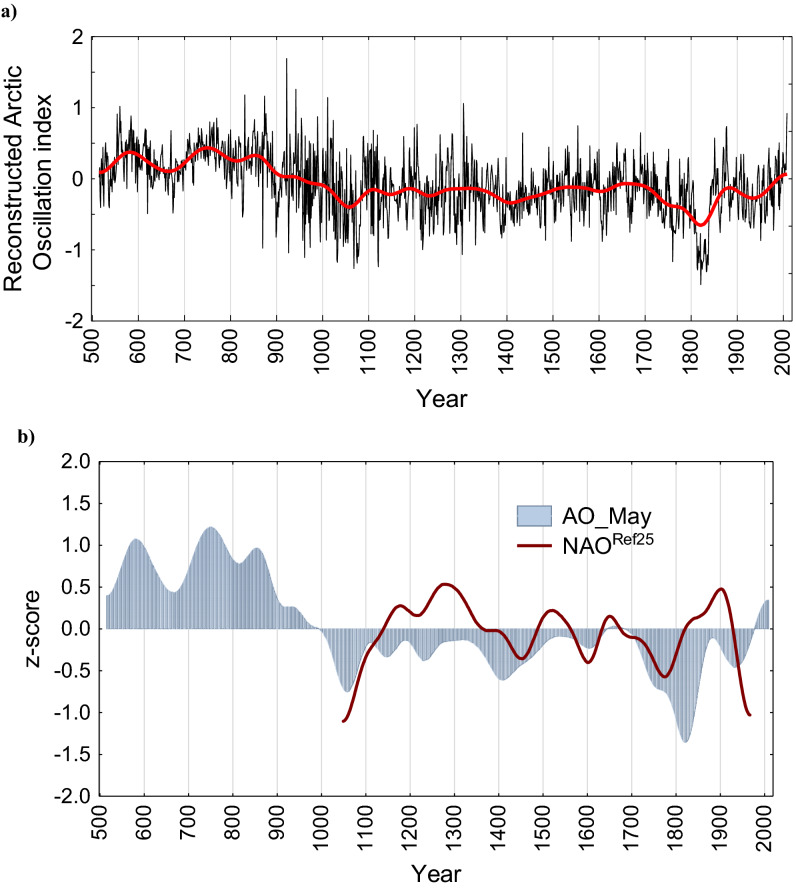
Annually-resolved reconstructed Arctic Oscillation (AO) index in May inferred from δ^18^O tree-ring cellulose (**a**) and smoothed by a 101-year Hamming window AO index in May in comparison with a proxy-based North Atlantic Oscillation (NAO)^25^ (**b**).

Significant correlation between AO index in May and δ^18^O_cell_ (r = 0.46, *P* < 0.05) for the period 1969–2009 CE and for the period (r = 0.26, *P* < 0.05) 1948–2009 were found (Fig. 2b, Supplementary Fig. S3) and based on the multiple linear regression analysis AO index in May was reconstructed (see Eq. (3), Fig. 4a).

Positive phases of the AO in May during the twentieth–twenty-first centuries were revealed. However, during the modern twentieth–twenty-first centuries the AO fluctuations do not exceed the amplitude of the second part of the first millennia and the medieval time (ninth–tenth centuries).

AO positive (AO+) phases in May (Fig. 4a) bring along warm and often dry conditions (Fig. 3). AO+ show higher fluctuation rates and amplitude during early Medieval Period compared to the recent one. A comparison with a proxy-based reconstructed NAO^25^ (Fig. 4b) showed significant correlation with AO in May (r = 0.38, p < 0.0001) for the period 1049–1969 CE. The chronologies agreed significantly mainly during the Little Ice Age (LIA, 1350–1800 CE) a positive anomaly during Late Antique Little Ice Age (LALIA, 516–600), early Medieval Climatic Anomaly (MCA, 700–1000 centuries) are well recorded in seasonal AO reconstructed index (Fig. 4b), while a divergence is apparent for the most recent part of the record.

Starting from the twentieth century a clearly increasing trend of the AO+ towards twenty-first century is observed. Along with AO+ a rapid decrease in precipitation is recorded in the July precipitation reconstruction (Fig. 3). A significant reduction of precipitation (> 80%) from the averaged mean and increasing summer temperature (daily maximum > 30 °C) correspond with extremely dry phases in July (> 3σ) in 1717, 1732, 1957, 1979, 1980, 2008, 2009 CE (Table 1). A positive AO phase during 2000–2009 CE was detected, which was opposite to the negative phase in the nineteenth century (1810–1820 CE) (Table 1).

The most pronounced negative phase of the AO was revealed during the past millennia (Fig. 4a,b, Table 1), which corresponded with major volcanic eruptions with a volcanic explosivity index (VEI ≥ 4). For example, Unknown or El Chichon (536, 541–542 CE, VEI ≥ 7), Samalas (1257 CE, VEI ≥ 7), La Soufrière 1812 CE and Mayon 1814 CE (VEI ≥ 4), Tambora (1815 CE, VEI ≥ 7), Galunggung (1822 CE, VEI ≥ 5), Cosiquina 1835 CE (VEI ≥ 5), Krakatoa 1883 (VEI ≥ 6) eruptions, which led to abnormally cold years. Some specific years (537–542, 1259, 1814–1815, 1818–1820, 1822–1828, 1831, 1834–1838 CE) recorded not only cold but also extremely wet conditions (Table 1).

### Spatial correlation analyses vs. climatic parameters

**Figure 5:**
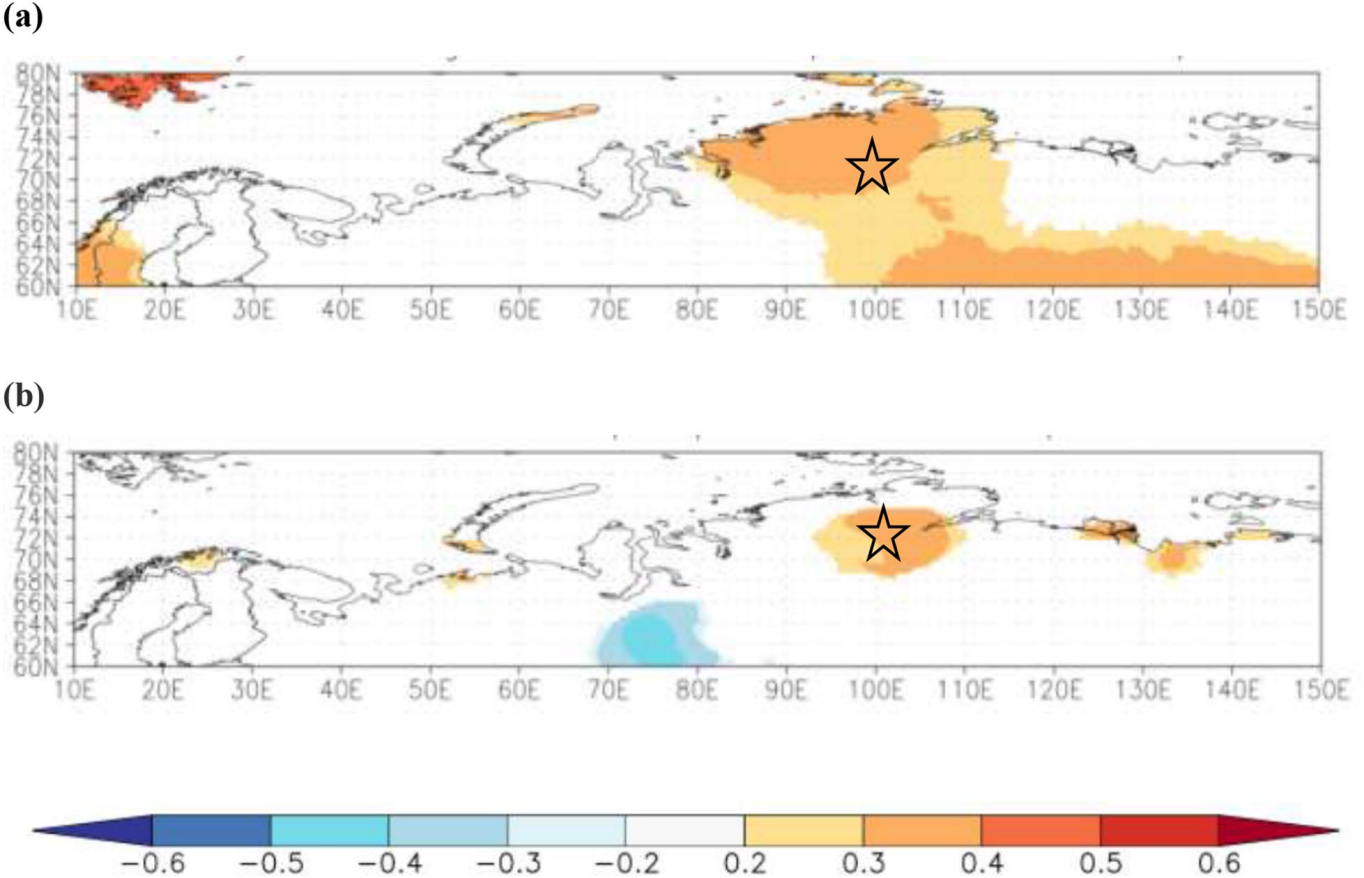
Spatial correlation coefficients computed between (**a**) reconstructed Arctic Oscillation (AO) indexes in May vs. CRU TS4.04 (*P* < 0.01) averaged May–July air temperature and (**b**) reconstructed July precipitation vs. CRU TS4.04 (*P* < 0.01) gridded July precipitation computed for the period 1969–2009 CE (*P* < 0.1) within the 60°–80° N, 10°–150° E. Scale represents a range from negative (− 0.6, blue color) to positive (0.6, red color) values. Star indicates the study site at the Taimyr Peninsula (70° N, 102° E). Climate explorer (KNMI climexp.knmi.nl) was used for plotting a grid net 0.5° within 60°–80° N and 10°–150° E.

Spatial correlation analyses showed a positive significant correlation between reconstructed AO in May and CRU TS4.04 maximum May–July air temperature (Fig. 5a) and July precipitation (Fig. 5b) (*P* < 0.01) computed for the common period of observation (1969–2009 CE) for both proxies.

The AO index in May correlated significantly with the May–July air temperature and shows a wide distribution from 80° N to 60° N, and from 95° E to 150° E (Fig. 5a) compared to the local distribution of July precipitation (Fig. 5b).

## Discussion

Oxygen isotopes in Siberian tree rings record not only a temperature signal^15–17,23,36,37^ but also capture information about air humidity and water origin, showing teleconnections with the Arctic Oscillation^16,22^ or North Atlantic Oscillation (NAO)^25,27^ via precipitation patterns. For instance, reconstructed NAO^25^ based on 48 proxy data with annual resolution distributed around the Atlantic Ocean showed similarities to our AO May reconstruction. Our AO reconstructed data agreed with NAO^25^ mainly during Little Ice Age (LIA, 1300–1800), emphasizing cold and dry conditions, while there was a disagreement for the recent decades. The Western part of Eurasian subarctic, which include Scandinavian regions, is strongly affected by internal variation and impact of North Atlantic Ocean, which brings wetter climate conditions during the recent period similar to the medieval time^27^.

In the Siberian north, the Medieval Warm Period during the tenth century was warmer but wetter compared to the recent period derived from our results. This is opposite to the northeastern part of Siberia, where recent atmospheric drought events significantly increased compared to the past^21^. Earlier warming in springs lead to earlier snowmelt^1^, developing a water shortage for trees by increasing the evapotranspiration in the Siberian subarctic^21^. Early warming caused by AO+ in May along with lack of precipitation and ground water availability (active soil layer at the top of 10 cm is still frozen) at the beginning of the growing season (late May–early June) in Taimyr Peninsula increases drought stress for larch trees. Recent severe fire periods were reported to occur early June–late July with a fire duration of 1.5 months^38^. Kharuk et al.^5^ showed that at high-latitude sites (> 70° N) extensive fires occurred in 1700–1999 CE based on fire scars and data of tree natality. This is in line with our July precipitation reconstruction, which indicates the development of drought, supporting the hypothesis that recent climatic warming will result in an increase of fire frequency at the Siberian subarctic. Furthermore, shifts of the growing period to earlier dates predicted by the process-based vegetation models^39^ will prolong the forest fire season.

We have therefore clear evidence that AO+ phases further promote the frequency of forest fires and fire spreading in boreal forests. Recent studies by Kirdyanov et al.^8^ confirm that lack of water during modern dry summers as well as pre-growing season precipitation can lead to the development of drought for trees and increase the frequency of fires. A study by Kim et al.^7^ revealed extensive fires in inner-Asian region linked to preceding positive Arctic Oscillation over the recent decades. An increase of atmospheric drying over the Siberian north^21^ and fire activity over the past decades^5,6,8,31,34,38^ can lead to long-term ecological consequences, where the main trigger are changes in the atmospheric circulation processes.

The increase in drought events may have started in the eighteenth century already, reaching a maximum and unprecedented values in twenty-first of century as shown with the July precipitation reconstruction. AO reconstruction based on the tree rings only^40^ suggests a strong positive phase of AO in the twentieth century, which is in line with our study. Recent drought anomalies were recorded also in other regions, like in European Alps and Central Europe^41,42^, however, not so pronounced compared to the Siberian subarctic regions. This can be explained by the impact of NAO index^43^, which responds more to a regional component and variability, than to the hemispheric one.

Our findings are consistent with an increased frequency of Siberian forest fires over the past decades in Central Siberia, very likely caused by the ongoing reduction of summer precipitation and triggered by a positive phase of the Arctic Oscillation in May.

## Material and methods

### Study site

Our study site is situated on the eastern part of the Taimyr Peninsula (TAY), where samples from living larch trees and rest of the stem wood on the ground surface were collected (TAY, 70–72° N, 100°–103° E, 200–300 m asl) (Supplementary Fig. S1).

All wood samples, including subfossil wood were well preserved due to severe cold climatic conditions and permafrost. The maximum permafrost thawing depth is recorded in middle of August but does not exceed 40–60 cm in depth, depending on slope exposition and soil composition^44^.

The eastern part of the Taimyr Peninsula is represented by tundra forests, which include zonal lowland forest tundra, pre-tundra and sub-alpine and open forests^13^. Larch stands and sparse forests of Ary-Mas occupy floodplain and floodplain terraces in the middle reaches of the Novaya River. Wood vegetation is represented by Gmelin larch (*Larix gmelinii* Rupr. Rupr.). Larch trees growing at this site can reach an age of 600 years, which is represented by the generation that appeared at the turn of twelfth–thirteenth centuries and are found in fresh and flowing-wet habitats (Supplementary Fig. S1b,c). Larch forests are represented by single trees growing in an open place with a distance between trees 5–8 m. The tree crowns closeness is up to 0.5^45^. Tree increment cores were collected from the living larch trees (n = 20). A larger number of larch wood samples from preserved dead trees (n = 138) and subfossil wood (n = 27) was collected. Sampling was carried out at the present upper timberline in the Stow Ary-Mas (72° 28′ N) and at the present upper timberline at 200–300 m a.s.l. in the Kotuy River valley (70° 30′ N–71° 00′ N).

### Khatanga weather station observation

Khatanga weather station (WSG 84, 71° N, 102° E, 33 m asl, http://climexp.knmi.nl) is the only one climate station located near the study site with available measurements from 1966 to 2009 CE. According to the Khatanga station data, the average annual temperature is − 12.8 °C. The warmest month is July with the maximum temperature plus 12.6 °C. The amount of precipitation is up to 280 mm per year. The vegetation period is rather short and does not exceed 85–90 days (early June-middle/end of August). Precipitation data from the local Khatanga weather station were used for the regression analysis and climate reconstruction.

### Arctic oscillation (AO) index

The seasonal Arctic Oscillation (AO) index is the leading mode in empirical orthogonal function analysis of wintertime monthly mean sea level pressure anomalies and is characterized by a seesaw of atmospheric mass between middle (20–30° N) and high (60–90° N) latitudes. The AO is also referred to as the Northern Hemisphere annular mode (NAM). The summertime atmospheric pattern hints at the link between the winter and summer NAM patterns^46^. Seasonal AO index (SV NAM index)^46^ is available from https://www.bio.mie-u.ac.jp/kankyo/shizen/lab1/AOindex.htm for the period from 1948 to 2020 CE. Year-round monthly anomaly and seasonal variability correlates significantly (r = 0.89; p < 0.0001) from 1969 to 2009 CE^46^. The seasonal dataset was used for the climate analysis with newly developed stable carbon and oxygen isotope chronologies in tree rings for the common period 1948–2009 CE.

### Selection of samples

Cross-dated 42 wood samples from larch trees with pointer years marked with steel needles were selected for the stable carbon and oxygen isotope analyses based on the following criteria: (i) similar age patterns; (ii) no missing rings; (iii) visually healthy trees without damages and scars. The average age of trees used for the analysis was 300 years. The first 50 years were not used for the analysis due to the possible juvenile period^47^. At least four sub-samples for the overlapping periods were used.

### Stable carbon and oxygen isotope analyses

Each annual tree-ring was split manually using a scalpel under the Leica stereo microscope (Leica, Germany). Each tree-ring was put in a filter bag with the identification number for the cellulose extraction according to the standard inter-laboratory protocol^48^. Each extracted tree-ring cellulose sample was homogenized, dried and weighted (0.2–0.3 mg) into the tin capsule for the ^13^C/^12^C analysis and into the silver capsule (0.5–0.8 mg) for the ^18^O/^16^O analysis. An isotope ratio mass spectrometer delta-S (Finnigan MAT, Bremen, Germany) linked to two elemental analyzers (EA-1110 Carlo Erba, Italy) via a variable open split interface (CONFLO-II, Finnigan MAT, Bremen, Germany) was used. The ^13^C/^12^C was determined by combustion and the ^18^O/^16^O by pyrolysis.

Samples for the periods 1110–1250, 1280–1630, 1670–1795 CE, were analyzed with a vario-PYRO cube (Elementar, Hanau, Germany) via thermal decomposition at 1450 °C and conversion to CO under O_2_ exclusion in helium. This system was linked to an IRMS (Delta plus XP, Thermo Finnigan, Bremen, Germany).

Both systems the EA-IRMS and the PYRO cube yielded very similar precisions (± 0.2‰) and values from two instruments were in high agreements^49^.

### Atmospheric δ^13^C of CO_2_ correction

Correction of δ^13^C_cell_ is necessary because the combustion of fossil fuels and biomass has resulted in a decrease of δ^13^C of the atmospheric CO_2_ over the last 150 years. By calculating the differences for each year to the pre-industrial value (1850 CE) for δ^13^C of atmospheric CO_2_ obtained from ice cores and direct atmospheric measurements at the Mauna Loa Observatory, Hawaii^50^ we subtracted these differences from the raw stable carbon isotope series from tree rings for each year. We did not apply any other corrections for the industrial period and back in time.

### Statistical analysis

To determine which climatic parameter or combination of climatic parameters impact stable carbon and oxygen isotopes in tree rings we applied multiple linear regression analyses.

Statistical characteristics such as Pearson correlation coefficient (r), reduction of error (RE), coefficient of efficiency (CE), and Durbin–Watson statistics (DW), coefficient of synchronicity (K_s_)^10^ were computed. Calibration and validation statistics are illustrated with their 2.5 and 97.5 percentiles and the reconstruction is given with its 95%-confidence intervals.

To reconstruct the climate back in time we applied a regression analysis, where climatic variables were the dependent variables, while stable isotope values were independent^9^ based on the equation (Eq. 1):

1 $${\text{Rec}}_{t} = a \cdot {\text{I}}_{t} + b + \varepsilon_{t} ,$$

where Rec_*t*_ is the reconstruction of the climate variable; e_*t*_ is the component of temperature variability, which is not explained by the variability of the stable isotope variation; I_t_ is the tree-ring parameter (δ^13^C_cell_ or δ^18^O_cell_), while *a* and *b* are intercept variables.

The variables resulting from the regression analyses are provided as equations (Eq. 2) for the period 1966–2009 CE for reconstruction of July precipitation based on the δ^13^C_cell_ chronology (Supplementary Fig. S2) and Eq. (3) is for reconstruction of the Arctic Oscillation (AO) index in May based on the δ^18^O_cell_ chronology for the period 1969–2009 CE (Supplementary Fig. S3). The regression variables for the calibration (1990–2009 CE) and verification (1969–1990 CE) periods are presented in the Supplementary Tables S2, S3.

2 $${\text{P}}_{{{\text{July}}}} = ( - 357.791 + ( - 16.723 \cdot \delta ^{{13}} {\text{C}}_{{{\text{cell}}}} )) + 0.15,$$

3 $$\text{AO}_{{{\text{May}}}} = \, ( - 7.075 + (0.320 \cdot \delta^{{{18}}} {\text{O}}_{{{\text{cell}}}} )) \, + \, 0.15.$$

Time series, Pearson correlation and multiple linear regression analyses were performed with the Statistica Software 13.0 (Statsoft Europe, Hamburg, Germany).

### Spatial correlation analysis

Climate explorer (World meteorological organization KNMI climexp.knmi.nl) was used for computing spatial correlations CRU TS4.04 (*P* < 0.01) between newly obtained annually-resolved climate reconstructions and climate parameters within 60°–80° N, 10°–150° E and a grid net 0.5°.

The Arctic Oscillation index in May and July precipitation reconstructions were correlated with a field of averaged May–July air temperature and July precipitation for the common period of instrumental measurements from 1966 to 2009 CE.

### Ethical approval

Relevant permits/permissions/licences were obtained: Wood sampling in the study site is complied with institutional and national guidelines and legislation. All methods were carried out in accordance with relevant guidelines and regulations.

## Supplementary Information

Supplementary Information.

## Data Availability

The datasets generated during and/or analysed during the current study will be available up on publication of the manuscript in Zenodo research data repository (10.5281/zenodo.5426606).
