# Supplementary material for: Spring arctic oscillation as a trigger of summer drought in Siberian subarctic over the past 1494 years
Source: Sci Rep. 2021 Sep 24;11:19010. doi: 10.1038/s41598-021-97911-2 (PMC8463678; doi:10.1038/s41598-021-97911-2)
Supplement: Supplementary file 1 — Supplementary Information. [file 41598_2021_97911_MOESM1_ESM.docx]

**Supplementary material:**

**Table S1.** Mean, maximum and minimum values for the δ^13^C_cell_ (a) and δ^18^O_cell_ (b) for the period from 516 to 2009 CE.

**(a)**

| **Year (CE)** | **Mean**  **(‰)** | **Maximum**  **(‰)** | **Minimum**  **(‰)** | **Standard deviation (SD)** | **Slope** |
| --- | --- | --- | --- | --- | --- |
| 516-600 | -25.52 | -23.60 | -26.75 | 0.67 | 0.56 |
| 601-700 | -24.93 | -24.96 | -25.92 | 0.36 | 0.14 |
| 701-800 | -25.32 | -23.94 | -25.34 | 0.32 | 0.35 |
| 801-900 | -25.35 | -23.07 | -26.44 | 0.56 | 0.34 |
| 901-1000 | -25.12 | -23.23 | -26.45 | 0.75 | -0.05 |
| 1001-1100 | -24.48 | -23.26 | -26.18 | 0.53 | -0.01 |
| 1101-1200 | -25.05 | -23.67 | -27.26 | 0.76 | 0.03 |
| 1201-1300 | -24.82 | -23.25 | -27.07 | 0.74 | 0.27 |
| 1301-1400 | -24.65 | -22.47 | -27.05 | 0.94 | 0.06 |
| 1401-1500 | -25.20 | -22.59 | -26.33 | 0.87 | -0.28 |
| 1501-1600 | -25.23 | -22.51 | -26.80 | 0.89 | 0.07 |
| 1601-1700 | -24.61 | -23.34 | -26.07 | 0.58 | 0.32 |
| 1701-1800 | -24.98 | -21.91 | -26.28 | 0.78 | 0.36 |
| 1801-1900 | -24.82 | -23.49 | -25.65 | 0.46 | 0.18 |
| 1901-2009 | -22.84 | -21.73 | -25.36 | 0.65 | 0.32 |
| 516-2009 | -24.86 | -21.73 | -27.26 | 0.80 | 0.06 |

**(b)**

| **Year (CE)** | **Mean**  **(‰)** | **Maximum**  **(‰)** | **Minimum**  **(‰)** | **Standard deviation (SD)** | **Slope** |
| --- | --- | --- | --- | --- | --- |
| 516-600 | 22.07 | 24.25 | 20.76 | 0.78 | 0.01 |
| 601-700 | 22.76 | 23.48 | 20.56 | 0.58 | -0.01 |
| 701-800 | 21.91 | 23.85 | 21.42 | 0.50 | -0.01 |
| 801-900 | 21.60 | 24.64 | 19.37 | 0.89 | -0.01 |
| 901-1000 | 20.63 | 25.89 | 19.05 | 1.14 | -0.01 |
| 1001-1100 | 22.41 | 24.55 | 18.67 | 1.26 | -0.01 |
| 1101-1200 | 22.36 | 23.51 | 18.73 | 0.93 | 0.01 |
| 1201-1300 | 21.02 | 23.63 | 19.31 | 0.97 | -0.01 |
| 1301-1400 | 21.23 | 24.35 | 19.15 | 0.89 | -0.01 |
| 1401-1500 | 20.94 | 23.34 | 19.65 | 0.59 | 0.01 |
| 1501-1600 | 21.67 | 23.59 | 19.61 | 0.69 | 0.01 |
| 1601-1700 | 22.68 | 23.34 | 19.60 | 0.76 | -0.01 |
| 1701-1800 | 20.93 | 22.78 | 18.90 | 0.78 | -0.01 |
| 1801-1900 | 20.98 | 23.56 | 18.12 | 1.25 | 0.02 |
| 1901-2009 | 22.75 | 24.01 | 19.63 | 0.83 | 0.01 |
| 516-2009 | 21.56 | 25.89 | 18.11 | 1.04 | -0.01 |

**Table S2.** Regression models for calibration and verification periods calculated between July precipitation Khatanga weather station and δ^13^C_cell_ chronology.

| Calibration | | | | | Verification | | | |
| --- | --- | --- | --- | --- | --- | --- | --- | --- |
| Period | R | R^2^ | F-criteria | DW | Period | R | R^2^ | К_s_ |
| 1966-2009 | -0.37 | 0.13 | 6.47  df=1.42  P<0.0001 | 1.95 |  |  |  |  |
| 1966-1987 | -0.49 | 0.24 | F=5.69  df=1.18  P<0.0001 | 2.19 | 1988-2009 | -0.54 | 0.29 | 0.60 |
| 1988-2009 | -0.54 | 0.29 | F=4.97  df=1.12  P<0.0001 | 2.63 | 1966-1987 | -0.49 | 0.24 | 0.70 |

**Table S3.** Regression models for calibration and verification periods calculated between Arctic Oscillation index in May and δ^18^O_cell_ chronology.

| Calibration | | | | | | Verification | | | |
| --- | --- | --- | --- | --- | --- | --- | --- | --- | --- |
| Period | R | R^2^ | F-criteria | DW | SE | Period | R | R^2^ | К_s_ |
| 1948-  2009 | 0.30 | 0.10 | 4.29  df=1.6  P<0.004 | 0.87 | 0.15 |  |  |  |  |
| 1948-  1978 | 0.32 | 0.14 | F=4.30  df=1.6  P<0.004 | 1.08 | 0.16 | 1979-2009 | 0.41 | 0.17 | 0.890 |
| 1979-  2009 | 0.41 | 0.26 | F=5.86  df=1.17  P<0.002 | 2.29 | 0.17 | 1948-1978 | 0.32 | 0.14 | 0.85 |

**(a)**


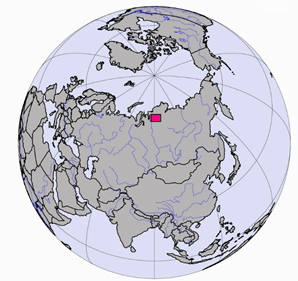


**(b) (c)**


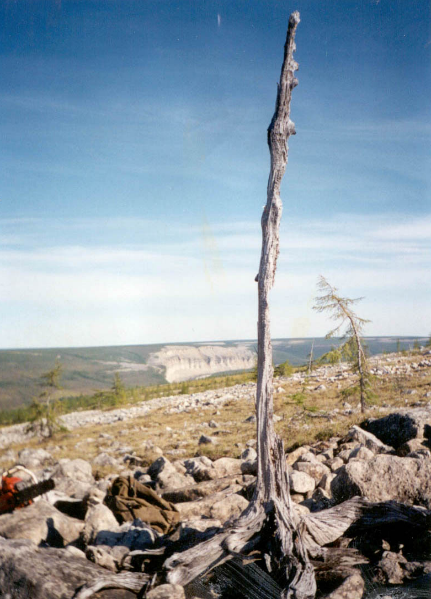

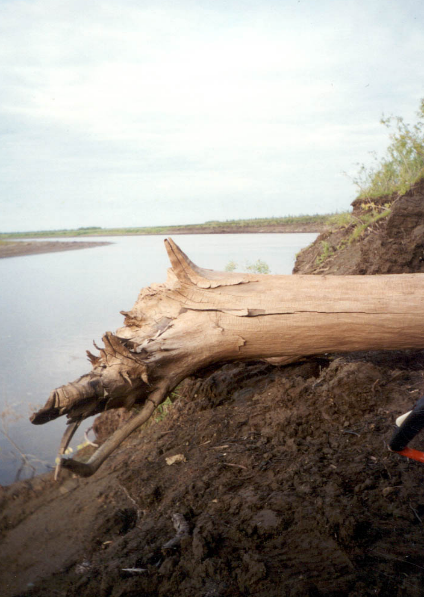


**Fig. S1.** Location of the study site (a) with old standing but dead Gmelin larch trees (*Larix gmelinii* Rupr. Rupr) (b) and subfossil trunk (c) laying on the ground surface from the study site in eastern Taimyr. Photos are part of the dendro archive stored at the Siberian Federal University made by M.M. Naurzbaev†. Map was created by using ESRI ArcGIS v.9 Software.

**Fig. S2.** Instrumental measurements of July precipitation from Khatanga weather station versus δ^13^C_cell_ for the period from 1966 to 2009 CE. Calibration (1990-2009 CE) and verification (1969-1990 CE) periods are separated by vertical red dotted line.

**Fig. S3.** Arctic oscillation (AO) index in May https://www.bio.mie-u.ac.jp/kankyo/shizen/lab1/AOindex.htm versus

δ^18^O_cell_ for the available period from 1948 to 2009 CE. Calibration (1979-2009 CE) and verification (1948-1978 CE) periods separated by vertical red dotted line.

urasia. Nature, 400(6740), 149–151. https ://doi.org/10.1038/22087aganov, E., Hughes, M., Kirdyanov, A., Schweingruber, F., & Silkin, P.

(1999). Influence of snowfall and melt timing on tree growth in subarctic

Eurasia. Nature, 400(6740), 149–151. https ://doi.org/10.1038/22087
